# Genomic Analysis and Delineation of the Tan Spot Susceptibility Locus *Tsc1* in Wheat

**DOI:** 10.3389/fpls.2022.793925

**Published:** 2022-03-23

**Authors:** Katherine L. D. Running, Aliya Momotaz, Gayan K. Kariyawasam, Jason D. Zurn, Maricelis Acevedo, Arron H. Carter, Zhaohui Liu, Justin D. Faris

**Affiliations:** ^1^Department of Plant Science, North Dakota State University, Fargo, ND, United States; ^2^USDA-Agricultural Research Service, Sugarcane Field Station, Canal Point, FL, United States; ^3^Department of Plant Pathology, North Dakota State University, Fargo, ND, United States; ^4^Department of Plant Pathology, Kansas State University, Manhattan, KS, United States; ^5^Department of Global Development, Cornell University, Ithaca, NY, United States; ^6^Department of Crop and Soil Sciences, Washington State University, Pullman, WA, United States; ^7^USDA-Agricultural Research Service, Cereal Crops Research Unit, Edward T. Schafer Agricultural Research Center, Fargo, ND, United States

**Keywords:** tan spot, wheat, *Triticum*, *Pyrenophora tritici-repentis*, Ptr ToxC, *Tsc1*, disease resistance

## Abstract

The necrotrophic fungal pathogen *Pyrenophora tritici-repentis* (*Ptr*) causes the foliar disease tan spot in both bread wheat and durum wheat. Wheat lines carrying the tan spot susceptibility gene *Tsc1* are sensitive to the *Ptr*-produced necrotrophic effector (NE) Ptr ToxC. A compatible interaction results in leaf chlorosis, reducing yield by decreasing the photosynthetic area of leaves. Developing genetically resistant cultivars will effectively reduce disease incidence. Toward that goal, the production of chlorosis in response to inoculation with Ptr ToxC-producing isolates was mapped in two low-resolution biparental populations derived from LMPG-6 × PI 626573 (LP) and Louise × Penawawa (LouPen). In total, 58 genetic markers were developed and mapped, delineating the *Tsc1* candidate gene region to a 1.4 centiMorgan (cM) genetic interval spanning 184 kb on the short arm of chromosome 1A. A total of nine candidate genes were identified in the Chinese Spring reference genome, seven with protein domains characteristic of resistance genes. Mapping of the chlorotic phenotype, development of genetic markers, both for genetic mapping and marker-assisted selection (MAS), and the identification of *Tsc1* candidate genes provide a foundation for map-based cloning of *Tsc1*.

## Introduction

*Pyrenophora tritici-repentis* (Died.) Drechs. (*Ptr*) is a necrotrophic homothallic ascomycete that causes the foliar disease tan spot in cultivated wheat, including common wheat (*Triticum aestivum* L., 2*n* = 6*x* = 42, AABBDD genomes), durum wheat [*Triticum turgidum* ssp. *durum* (Desf.) Husnot., 2*n* = 4*x* = 28, AABB genomes], and wild relatives (reviewed by Faris et al., 2013). Tan spot or yellow leaf spot, was first described as a minor pathogen in 1823 (Hosford Jr., 1982). Tan spot epidemics began in the 1970s, coinciding with the adoption of minimum tillage practices. Minimum tillage practices are believed to have caused an increase in disease incidence because *Ptr* overwinters on wheat residue, infecting crops the following season. Crop rotations and fungicide applications can reduce disease incidence and severity, but the most effective method for reducing disease incidence is through the development of genetically resistant varieties.

*Pyrenophora tritici-repentis* produces and secretes multiple necrotrophic effectors (NEs). The recognition of NEs by corresponding host sensitivity genes leads to a compatible interaction resulting in the development of necrotic and chlorotic lesions. The NEs and host sensitivity genes interact in an inverse gene-for-gene manner where the pathogen hijacks host defense pathways leading to necrotrophic effector triggered susceptibility (NETS; Liu et al., 2009; Friesen and Faris, 2010). These necrotic and chlorotic lesions reduce the photosynthetic area of the plant resulting in reduced kernel weight and grain number (Shabeer and Bockus, 1988).

Three host sensitivity gene-*Ptr* NE interactions have been characterized so far: *Tsn1*-Ptr ToxA, *Tsc2*-Ptr ToxB, and *Tsc1*-Ptr ToxC (reviewed by Faris et al., 2013). One host sensitivity gene, *Tsn1* (Faris et al., 2010), and two NE genes, *PtrToxA* (Ballance et al., 1996; Ciuffetti et al., 1997) and *PtrToxB* (Martinez et al., 2001), have been cloned. The *Tsn1*-Ptr ToxA interaction produces necrosis, whereas the *Tsc2*-Ptr ToxB and *Tsc1*-Ptr ToxC interactions produce chlorosis. *Ptr* isolates are classified into races depending on their virulence patterns on a set of host differentials (reviewed by Faris et al., 2013).

In addition to the inverse gene-for-gene interactions, five tan spot resistance genes have also been identified (reviewed by Faris et al., 2013) including a major dominant gene, *Tsr7*, that confers race-nonspecific resistance in both tetraploid and hexaploid wheat (Faris et al., 2020). The other tan spot resistance genes, *tsr2* (Singh et al., 2006), *tsr3* (Tadesse et al., 2006a), *tsr4* (Tadesse et al., 2006b), and *tsr5* (Singh et al., 2008), confer recessive resistance. It is therefore possible that they are recessive alleles of host sensitivity genes that interact with yet unidentified NEs (reviewed in Faris et al., 2013).

A quantitative trait locus (QTL, *QTsc.ndsu-1A*) associated with resistance to chlorosis induced by Ptr ToxC-producing isolates was first identified in the International Triticeae Mapping Initiative W-7984 × Opata 85 recombinant inbred line (RIL) population (Faris et al., 1997; Effertz et al., 2002). The same QTL was shown to coincide with Ptr ToxC sensitivity (Effertz et al., 2002), and the gene underlying sensitivity was designated *Tsc1*. Ptr ToxC was predicted to be a small non-ionic, polar, molecule that induces chlorosis on wheat varieties possessing *Tsc1*.

A QTL designated *QTs.zhl-1A* was mapped to chromosome arm 1AS in two RIL populations (Kariyawasam et al., 2016; Liu et al., 2017) corresponding to the position of *QTsc.ndsu-1A*. In a RIL population derived from the cross Louise × Penawawa (LouPen; Carter et al., 2020), *QTs.zhl-1A* was associated with diseased caused by race 1, race 3, and AR CrossB10 *Ptr* isolates and explained up to 22% of the phenotypic variation (Kariyawasam et al., 2016). F_1_ plants from the same cross exhibited chlorosis after inoculation with the race 3 isolate 331-9, indicating that the chlorosis was conferred by a dominant susceptibility gene as opposed to the lack of a dominant resistance gene. In the LMPG-6 × PI 626573 (LP) RIL population, *QTs.zhl-1A* was associated with susceptibility explaining up to 27% of the variation in disease.

Additional QTL corresponding to the *Tsc1* region have been identified in many hexaploid populations including, but not limited to, the biparental populations TA161-L1 × TAM105 (Kalia et al., 2018), IGW2547 × Annuello (Shankar et al., 2017), and Ernie × Betavia (Li et al., 2011), and a MAGIC population derived from Event, BAYP4535, Ambition, Firl3565, Format, Potenzial, Bussard, and Julius (Stadlmeier et al., 2019). A meta-QTL analysis identified two meta-QTLs in the *Tsc1* region. However, they likely both correspond to *Tsc1* (Liu et al., 2020). QTL in the *Tsc1* region have also been identified in durum wheat. In a worldwide collection of durum wheat, a recent evaluation using a Ptr ToxC-producing isolate revealed a QTL, likely corresponding to *Tsc1*, on the short arm of chromosome 1A (Galagedara et al., 2020).

**Figure 1:**
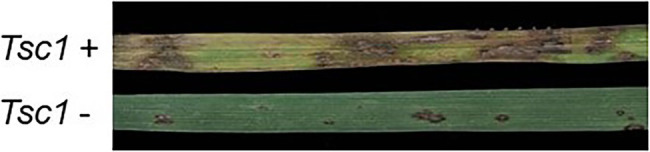
Leaves of wheat genotypes with *Tsc1* (top) and without *Tsc1* (bottom) inoculated with a *Pyrenophora tritici-repentis* (*Ptr*) ToxC-producing isolate.

Wheat lines containing the *Tsc1* gene exhibit a large amount of chlorosis resulting in severe tan spot susceptibility when infected with Ptr ToxC-containing isolates (Figure 1). Our long-term goal is to clone the *Tsc1* gene using a map-based approach to gain a better understanding of the *Tsc1*-Ptr ToxC interaction at the molecular level. Toward this goal, the objectives of the current research were to: (1) develop molecular markers and saturated genetic linkage maps of the genomic region containing the *Tsc1* gene, (2) define and characterize the genetic and physical interval containing the *Tsc1* locus, and (3) identify candidate genes for *Tsc1* in the wheat reference genome sequence. Achievement of these objectives provides a strong foundation for launching the next phase of objectives toward map-based cloning of *Tsc1*.

## Materials and Methods

### Plant Materials

The LouPen and LP biparental populations were used to map newly developed markers within the *Tsc1* region. Louise and LMPG-6 exhibit extensive chlorosis when inoculated with Ptr ToxC-producing isolates because they carry the dominant *Tsc1* allele, whereas Penawawa and PI 626573 are free of chlorosis when inoculated with the same isolates because they harbor the recessive *tsc1* allele (Kariyawasam et al., 2016; Liu et al., 2017). The LouPen population consists of 188 RILs and was originally developed to map stripe rust resistance derived from Louise (Carter et al., 2009). The LP population consists of 240 RILs and was originally developed to map stem rust Ug99 resistance in PI 626573 (Zurn et al., 2014). Sixteen hexaploid varieties were genotyped with markers closely linked to *Tsc1* to test the usefulness of markers for marker-assisted selection (MAS; Table 1).

**Table 1:** Allelic state and corresponding references of hexaploid genotypes evaluated with markers developed in this research and linked to *Tsc1*.

Genotype	*Tsc1* allele	Reference
Opata 85	*Tsc1*	Faris et al., 1997
Louise	*Tsc1*	Kariyawasam et al., 2016
LMPG-6	*Tsc1*	Liu et al., 2017
6B365	*Tsc1*	Lamari and Bernier, 1989
Kulm	*Tsc1*	Effertz et al., 2002
Trenton	*Tsc1*	Effertz et al., 2001
Ning 7840	*Tsc1*	Sun et al., 2010
W-7984	*tsc1*	Faris et al., 1997
Penawawa	*tsc1*	Kariyawasam et al., 2016
PI 626573	*tsc1*	Liu et al., 2017
Glenlea	*tsc1*	Lamari and Bernier, 1989
6B662	*tsc1*	Lamari et al., 1995
Salamouni	*tsc1*	Lamari et al., 1995
Chinese Spring	*tsc1*	Tadesse et al., 2006a
Erik	*tsc1*	Singh and Hughes, 2006
Katepwa	*tsc1*	Lamari et al., 1995

### Inoculations and Disease Evaluation

The LouPen and LP populations were inoculated with the Ptr ToxC-producing race 3 isolate 331-9 in Kariyawasam et al. (2016) and Liu et al. (2017), respectively. Although previously unreported, data on the presence and absence of chlorosis induced by isolate 331-9 were collected, and that data were used here to map chlorosis induction as a qualitative trait representing the *Tsc1* locus in both populations.

### Marker Development and *Tsc1* Mapping

The LouPen and LP populations were previously genotyped with the wheat 9 K iSelect Assay BeadChip (Cavanagh et al., 2013), and whole genome maps were assembled (Zurn et al., 2014; Kariyawasam et al., 2016). Several methods were used to develop and/or identify additional markers within the *Tsc1* genomic region of chromosome 1A. First, simple sequence repeat (SSR) markers previously mapped and known to detect loci on chromosome arm 1AS were identified from the Graingenes database.^1^

Second, contextual sequences of SNP markers derived from the 9 and 90 K arrays known to map to the short arm of chromosome 1A were used as queries in BLASTn searches of either Chinese Spring survey sequences (International Wheat Genome Sequencing Consortium (IWGSC), 2014), the Chinese Spring reference genome v1.0 (International Wheat Genome Sequencing Consortium (IWGSC) et al., 2018), or the wild emmer wheat genome sequence of Zavitan (Avni et al., 2017). The corresponding survey sequences and approximately 10 kb segments of the Chinese Spring and Zavitan genome sequences encompassing the SNP BLAST hits were then subjected to searches for SSRs using SSRIT^2^ and gene-like or low-copy DNA features by using the survey sequence or extracted genome segment sequence as a query in BLASTx searches against the NCBI non-redundant database.^3^ SSRs and gene-like features were used to develop SSR and sequence-tagged site (STS) markers, respectively, and primers were designed using Primer 3 (Rozen and Skaletsky, 1999).

Third, a genome-wide association study of tan spot resistance in durum wheat (Galagedara et al., 2020) revealed a genotype-by-sequencing (GBS) marker on chromosome arm 1AS associated with reaction to the Ptr ToxC-producing isolate Pti2 and was therefore likely associated with *Tsc1*. We used the sequence of this GBS marker to develop a semi-thermal asymmetric reverse PCR (STARP) marker (Long et al., 2017) to map the locus in the LouPen and LP populations.

All markers were amplified *via* PCR and electrophoresed on 6% nondenaturing polyacrylamide gels. Gels were stained with Gelred™ nucleic acid stain (Biotium Corporate, Hayward, CA, United States), and scanned with a Typhoon 9410 or FLA 9500 variable mode imager (GE healthcare Biosciences, Waukesha, WI, United States). Genetic linkage maps were constructed in MapDisto v2.1.7 (Heffelfinger et al., 2017) as described in Faris et al. (2014). Maps were visualized in MapChart 2.32 (Voorrips, 2002). All PCR primers used for the identification of markers in this research are listed in Supplementary Table 1.

### Identification of Candidate Genes

The closest flanking markers to *Tsc1* (*fcp730* and *fcp734*) in the LouPen genetic map were used to identify candidate regions in the Chinese Spring v2.1 assembly (Zhu et al., 2021). High- and low-confidence annotated genes in the Chinese Spring v2.1 reference assembly were considered for analysis of protein domains (accessed December 7, 2021). Conserved protein domains of the annotated genes were identified by searching the Pfam database.^4^ Genes less than 500 bp long or those with no Pfam hits more significant than 1 × 10^−5^ were considered pseudogenes or gene fragments and were excluded from further analysis.

## Results

### Saturation Mapping of the *Tsc1* Locus

In the first LouPen genetic map, *Tsc1* mapped distal to the 9 K SNP markers *IWA4643*, *IWA414*, *IWA3680*, and *IWA1388*, thus placing *Tsc1* within the first 15.2 Mb of the Chinese Spring v2.1 chromosome 1A short arm. Testing of SSR markers previously mapped to chromosome 1AS in other wheat mapping populations identified six markers polymorphic between Louise and Penawawa (Supplementary Table 1). Amplicon sequence analysis revealed the SSR markers *gpw7072* and *psp2999* targeted the same locus (data not shown). Once these six SSR markers were added to the genetic linkage map, the *Tsc1* region was narrowed to approximately the first 5 Mb of the physical map. At this point, all markers mapped proximal to *Tsc1*, and more markers needed to be developed, particularly distal to *Tsc1*, to delineate the *Tsc1* region.

**Figure 2:**
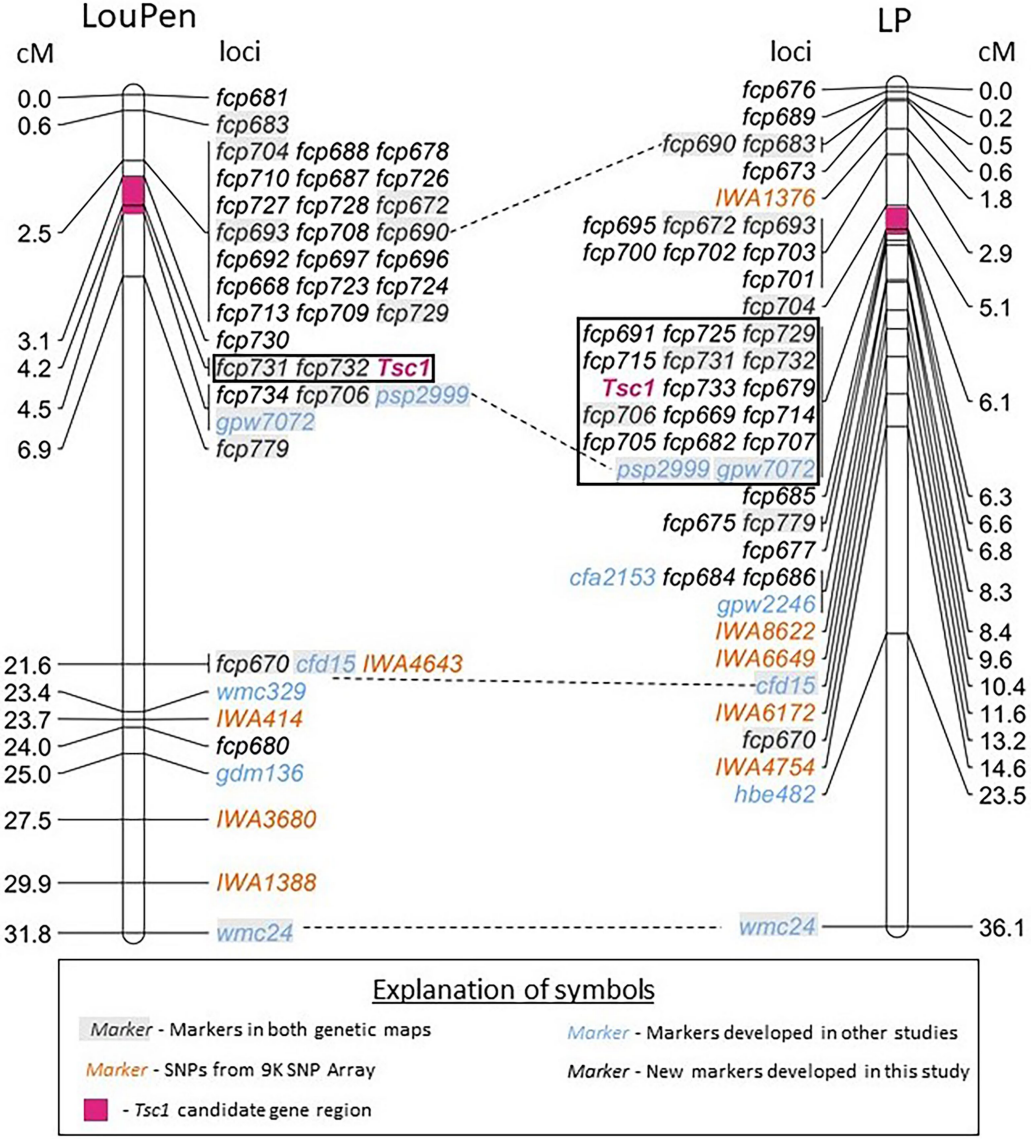
Saturation maps of the *Tsc1* region developed in Louise × Penawawa (LouPen) and LMPG-6 × PI 626573 (LP) populations. The LouPen genetic map is on the left and the LP genetic map is on the right. Loci mapped are listed on the right of the LouPen genetic map and the left of the LP genetic map. Opposite the loci, the genetic distances are displayed in centiMorgans (cM). Markers in orange are SNP markers from the wheat 9 K iSelect Assay BeadChip. Markers in black are simple sequence repeat (SSR) markers designed in this study. Blue markers were designed in other studies. Dashed lines connect markers mapped in both populations. The black outlined rectangle indicates the loci cosegregating with *Tsc1*. The pink shaded portion of the chromosome represents the candidate gene region in each population.

Prior to the availability of the whole genome reference sequence of the hexaploid wheat cultivar Chinese Spring, SNPs from the 9 and 90 K SNP arrays known to map to chromosome 1AS were used to identify Chinese Spring survey sequences. Twelve STS markers and two SSR markers designed from the survey sequences were polymorphic and mapped in the LouPen population (Supplementary Table 1). An additional three SSRs and one STS were designed from the Zavitan genome assembly as well as 10 SSRs from the Chinese Spring reference v1.0. Some of the newly designed markers mapped distal to *Tsc1* and further delineated the *Tsc1* region. *Tsc1* cosegregated with two markers, and the candidate gene region based on the genetic map constructed in the LouPen population was 184 kb. In total, the LouPen genetic map spanned 31.8 centiMorgans (cM) with 42 loci and had a marker density of 1.32 markers/cM (Figure 2).

The initial genetic map of the LP population placed *Tsc1* within a ~7.2 Mb region of the short arm of chromosome 1A between the 9 K SNP markers *IWA1376* and *IWA8622*. Seven previously mapped SSR markers were polymorphic between LMPG-6 and PI 626573, including four that were included in the LouPen genetic map. The inclusion of these seven markers on the LP genetic linkage map delineated the *Tsc1* region to 3.9 Mb on the physical map.

To reduce the candidate gene region further, additional markers were designed in the same manner as they were for mapping in the LouPen population. Fourteen STS and five SSR markers designed from the Chinese Spring survey sequences and eight SSR and two STS markers derived from the Zavitan genome assembly were mapped in the LP population (Supplementary Table 1). An additional five SSR markers designed from the Chinese Spring reference v1.0 were added to the LP genetic map. These additional STS and SSR markers reduced the candidate gene region to approximately 1 Mb, an order of magnitude larger than the candidate gene region defined by mapping in the LouPen population. The LP map consisted of 47 loci spanning 36.1 cM, which gives a marker density of 1.30 markers/cM (Figure 2).

Recombination rates were compared between the LP and LouPen populations within the mapped regions to determine which population delineated the *Tsc1* locus to the smallest genomic region, or if a composite of the two maps could be used to define the *Tsc1* locus to a smaller region. The most distal and proximal markers in common between the two maps were *fcp683* and *wmc24*, respectively. The region defined by these markers encompassed 26.3 Mb on the Chinese Spring v2.1 reference genome, and it spanned 31.2 and 35.5 cM of genetic distance in the LouPen and LP populations, respectively. Therefore, the recombination rate across this region was higher in the LP population (1.35 cM/Mb) compared to the LouPen population (1.19 cM/Mb).

Comparison of recombination rates in the vicinity of the *Tsc1* locus revealed a different scenario. The markers *fcp704* and *fcp779*, which were the two markers in common to both maps that detect recombination events most closely flanking *Tsc1* on the distal and proximal sides, respectively, were separated by 4.4 cM on the LouPen map and 1.5 cM on the LP map (Figure 2). Unfortunately, the amplicon sequence for *fcp704* was not present in the Chinese Spring v2.1 genome making it impossible to determine the physical distance between these common flanking markers. The next closest marker on the distal side of *Tsc1* common to both maps and present in Chinese Spring was *fcp693*. The genetic distances between *fcp779* and *fcp693* in the LouPen and LP populations were 4.4 and 3.7 cM, respectively. The physical distance between these two markers in the Chinese Spring reference genome was 5.7 Mb, which translates to 0.77 cM/Mb in the LouPen population and 0.64 cM/Mb in the LP population. Therefore, the recombination frequency near the *Tsc1* locus was higher in the LouPen population compared to the LP population.

**Figure 3:**
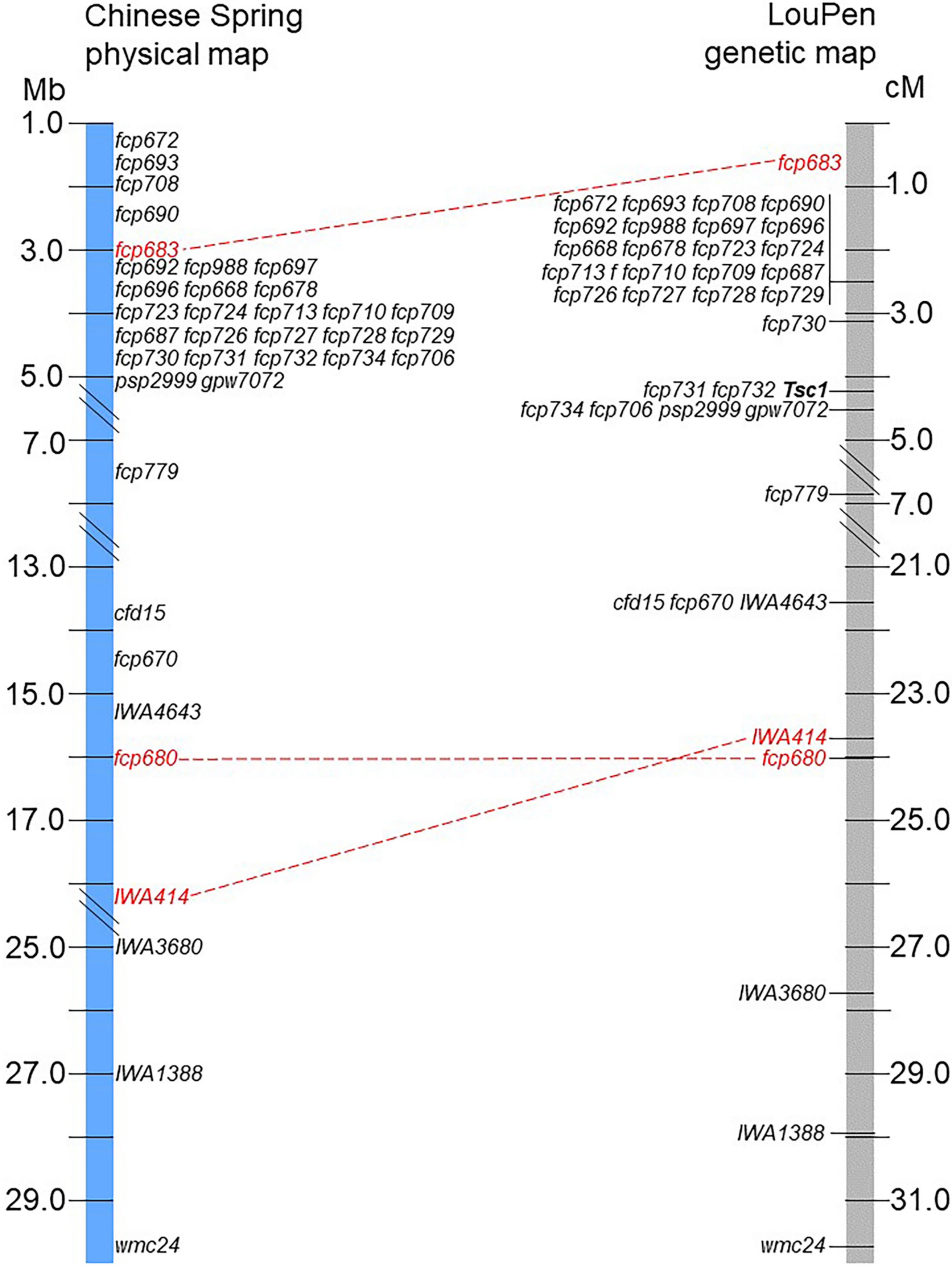
Comparison of the physical and genetic order of markers. The LouPen genetic map is on the right and the Chinese Spring v2.1 physical map is on the left. Markers in red font connected by red dashed lines are not colinear. All other markers are colinear.

The genetic order of the markers in LouPen was compared to the physical order in the Chinese Spring v2.1 reference genome due to the higher genetic resolution near *Tsc1* (Figure 3). There were two instances of non-collinearity. Firstly, marker *fcp683* mapped more distal in LouPen than its physical position, which would place it within the markers cosegregating at 2.5 cM. On the proximal side of *Tsc1*, the markers *IWA414* and *fcp680* were inverted relative to their physical position. These minor inconsistencies between genetic and physical order of the markers are indicative of rearrangements in the Chinese Spring genome relative to Louise and Penawawa. However, the rearrangements do not encompass or alter the candidate gene region.

### Delineation of the Candidate Gene Region and Identification of Candidate Genes

**Figure 4:**
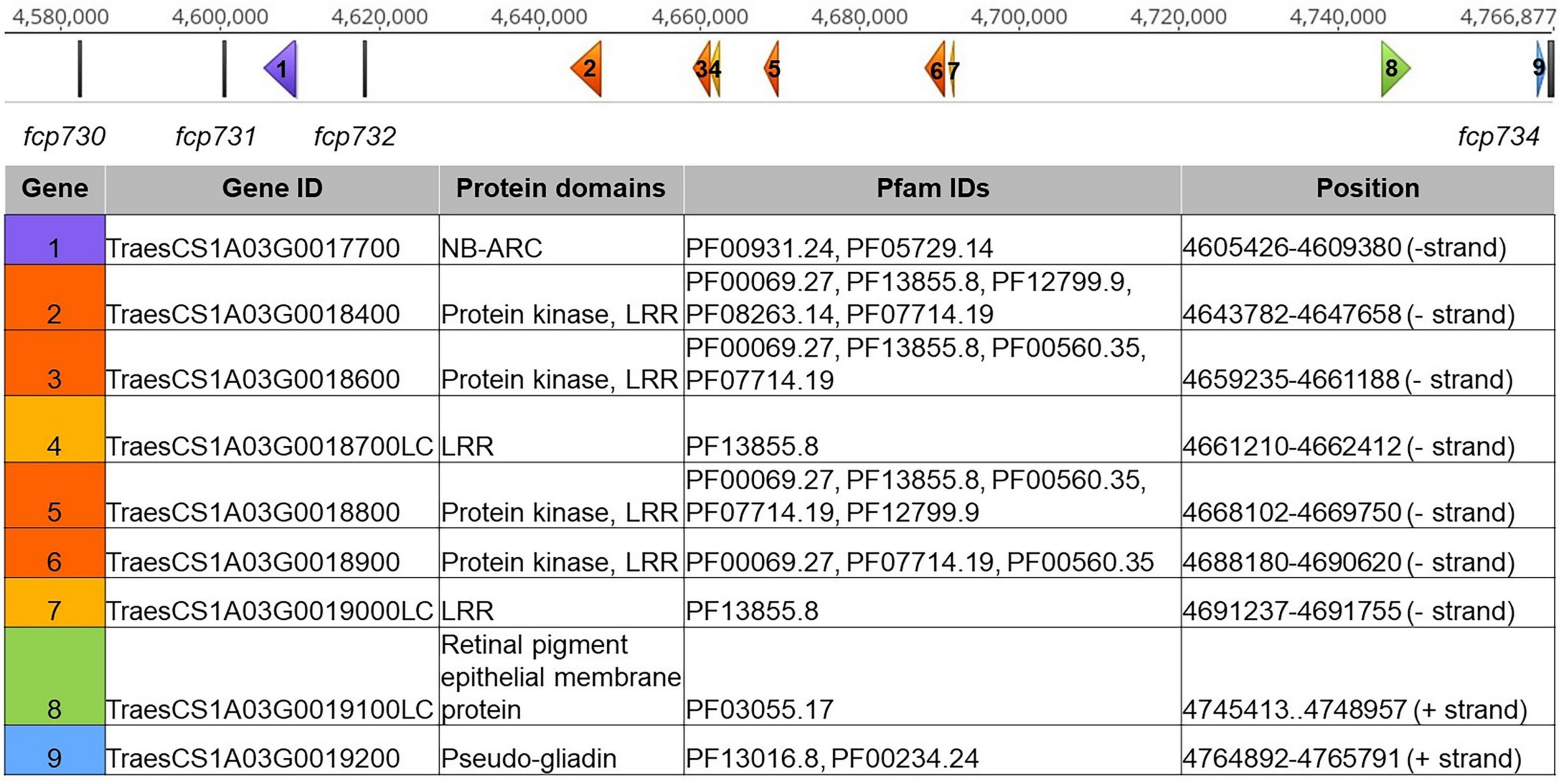
*Tsc1* candidate gene region in Chinese Spring reference genome v2.1. The scale on the top represents the physical position in base pairs. Genetic markers are displayed as vertical gray bars. Genes are displayed as arrows, labeled 1–5, corresponding to the genes in the table below. Genes with nucleotide binding and ARC (NB-ARC), protein kinase (PK) and leucine rich repeat (LRR), LRR, retinal pigment epithelial membrane, and gliadin domains are shown in purple, orange, yellow, green, and blue, respectively. Gene IDs, protein domains, Pfam IDs, and physical positions of each gene are included in the table.

In the LouPen population, the *Tsc1* candidate gene region was delineated by *fcp730* and *fcp734*, which were 1.4 cM apart (Figure 2). This region corresponded to approximately 184 kb in the Chinese Spring reference v2.1 genome (Figure 4). Two markers, *fcp732* and *fcp731* cosegregated with *Tsc1*, and they spanned just 17 kb.

The candidate gene region, delineated by *fcp704* and *fcp685*, was larger in the LP population. As *fcp704* is not in the Chinese Spring reference genome, the next closest marker, *fcp701*, was selected to delineate the candidate gene region to 3.9 Mb in the LP population. The 16 markers that cosegregated with *Tsc1* spanned a total of 967 kb in the Chinese Spring v2.1 reference genome.

Given this finding, the delineated region on the genetic map developed in the LouPen population was used to define the *Tsc1* candidate region and to identify candidate genes based on the Chinese Spring reference sequence (Figure 4). No genes were identified between the distal flanking marker *fcp730* and the markers *fcp731* and *fcp732*, which cosegregated with *Tsc1*. A gene containing nucleotide binding and ARC (NB-ARC) domains was identified between *fcp731* and *fcp732*. Between *fcp732* and the proximal flanking marker, *fcp734*, there were four protein kinase and leucine rich repeat (PK-LRR) domain-containing genes and two genes with only an LRR domain. Two additional genes within this segment included a gene with a retinal pigment epithelial membrane protein domain and a pseudo-gliadin gene. The former was considered a gene fragment as it did not contain a start codon. A large family of gliadins is known to exist on chromosome 1A in wheat, so it is not surprising that a pseudo-gliadin was identified. However, gliadins have not been shown to be involved in disease resistance or susceptibility, and therefore the pseudo-gliadin gene was not considered a candidate. In total, nine genes were identified in Chinese Spring and seven are considered candidates including one NB-ARC, four PK-LRRs, and two LRR domain-containing genes (Figure 4).

### Evaluation of Markers Closely Linked to *Tsc1*

To identify markers that could be potentially used for MAS of Ptr ToxC-insensitive lines, i.e., elimination of the dominant *Tsc1* allele, markers closely linked to the *Tsc1* locus were evaluated on a panel of hexaploid wheat lines on which phenotypic evaluations with Ptr ToxC-producing isolates has been conducted, and therefore the allelic status at the *Tsc1* locus is known (Table 1). The markers *fcp731* and *fcp732*, which cosegregated with *Tsc1* in the LouPen population, were selected for evaluation as well as *fcp729* and flanking markers *fcp730*, *fcp734*, and *psp2999*. Among the hexaploid lines evaluated, seven were resistant to chlorosis induced by Ptr ToxC-producing isolates of *Ptr*, and nine were susceptible and developed extensive chlorosis (Table 1).

**Figure 5:**
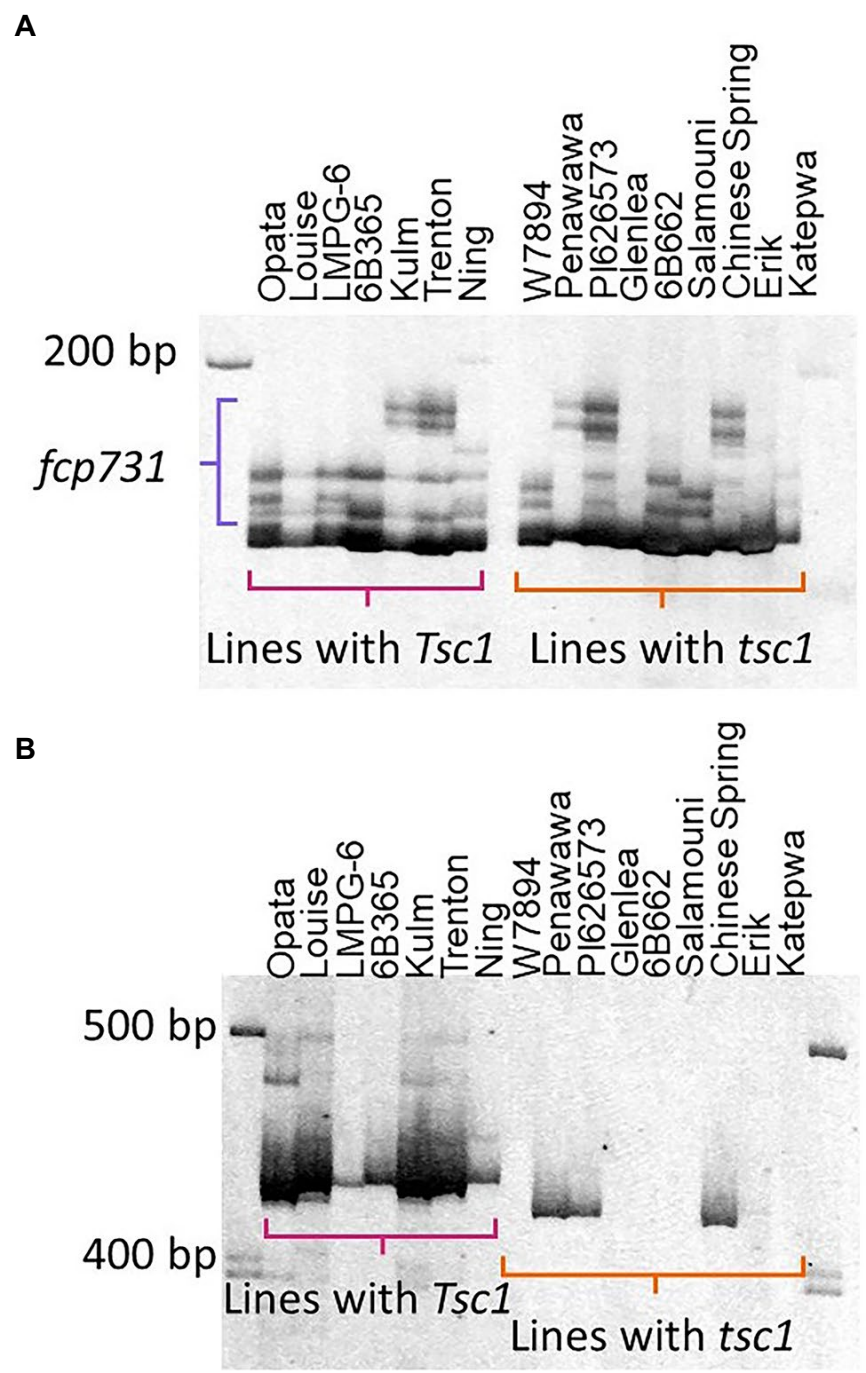
Evaluation of markers cosegregating with *Tsc1*. The polyacrylamide gel images of markers *fcp731* **(A)** and *fcp732* **(B)** run on lines with known sensitivity statuses (Table 1) are shown. Horizontal brackets in pink and orange denote amplicons in lines with *Tsc1* and *tsc1*, respectively. The primary amplicon was scored for marker *fcp732* **(B)**. The amplicons denoted by the purple bracket were scored for marker *fcp731* **(A)**.

Analysis of amplified fragments for these six markers revealed that no marker allele was associated with the allelic state of *Tsc1* (Figure 5). The best association was with *fcp732* where five out of nine resistant lines had null marker alleles.

## Discussion

The *Tsc1*-Ptr ToxC interaction plays a significant role in tan spot development in both hexaploid and tetraploid backgrounds (Galagedara et al., 2020; Liu et al., 2020). Identification of *Tsc1* would allow further characterization of the *Tsc1*-ToxC interaction, including the molecular mechanisms underlying the interaction. While there are extensive tan spot QTL analyses, *Tsn1* is the only host sensitivity gene cloned to date (Faris et al., 2010).

The markers in this study delineate *Tsc1* to 1.2 cM in the LP population and 1.4 cM in the LouPen population. Interestingly, *Tsc1* cosegregated with 16 markers in the LP population that spanned 967 kb, demonstrating reduced recombination in the LP population relative to the LouPen population. Although relative recombination rates indicate that future mapping would likely be more successful in the LouPen population than the LP population, candidate gene analysis indicates that future high-resolution mapping may be unnecessary.

Candidate gene analysis revealed seven genes with domains common among resistance genes, which are often hijacked by necrotrophic pathogens to induce disease (Faris and Friesen, 2020). The first candidate gene on the distal side of the candidate gene region is an NB-ARC domain-containing gene. NB-ARC proteins are a subclass within the protein super family “signal transduction ATPases with numerous domains” (STAND; Danot et al., 2009). Often, NB-ARC domain-containing proteins have an additional domain, like an LRR domain that acts as a sensor. Although the NB-ARC candidate gene in this region does not contain an LRR domain, it is still possible that it is involved in the recognition of Ptr ToxC or the signal transduction that leads to programmed cell death.

Between the markers that cosegregated with *Tsc1* and the proximal flanking marker, there were four PK-LRR domain-containing genes and two genes containing only an LRR domain predicted in the Chinese Spring v2.1 reference genome. We consider the four PK-LRR genes to be the stronger candidates. The receptor-like protein kinase family can recognize signal peptides, either directly or indirectly, dimerize, and initiate signaling pathways through phosphorylation cascades (Butenko et al., 2009). All four PK-LRR genes have transmembrane domains and therefore, are likely cell surface proteins. Genetic mutation analysis of these candidate genes is underway to determine which of the seven candidates, if any, is *Tsc1*.

It is promising that many of the genes identified in the candidate gene region of Chinese Spring contain domains identified in previously characterized necrotrophic susceptibility genes (reviewed by Faris and Friesen, 2020). In the wheat-*Parastagonospora nodorum* pathosystem, three necrotrophic susceptibility genes have been cloned. The first, *Tsn1*, is also the tan spot susceptibility gene that confers susceptibility to isolates producing Ptr ToxA. *Tsn1* has NB, PK, and LRR domains (Faris et al., 2010). The second cloned *P. nodorum* susceptibility gene, *Snn1*, is a wall-associated kinase. The third cloned *P. nodorum* susceptibility gene, *Snn3-D1*, is a protein kinase-major sperm domain-containing gene (Zhang et al., 2021). So far, all cloned necrotrophic susceptibility genes in wheat contain a PK domain. As such, we believe that the PK-LRR genes are stronger candidates for *Tsc1*.

Although the *Tsc1* candidates in the Chinese Spring genome are logical susceptibility genes, it is possible that an allele of *Tsc1* is not present in the Chinese Spring genome. Chinese Spring is not susceptible to Ptr ToxC-induced chlorosis, and therefore does not harbor a functional *Tsc1* allele. We are characterizing the 10+ wheat genomes to determine if any of the sequenced wheat varieties are susceptible to Ptr ToxC chlorosis. Combining genomic analysis of the gene content, gene alleles, and the phenotypes across the 10+ wheat genomes may allow us to further reduce the number of *Tsc1* candidate genes.

Analysis of markers closely linked to *Tsc1* on a set of genotypes with known sensitivity statuses revealed multiple alleles for each marker as well as multiple haplotypes, suggesting that *Tsc1* lies within a region of high recombination in natural populations. For example, marker *fcp731*, which cosegregated with *Tsc1*, had four alleles within the susceptible lines and five alleles within the resistant lines. This diversity in marker alleles was likely helpful in finding polymorphic markers to use in genetic mapping, but it are less useful in MAS. As such, it is not recommended that these markers be used to select resistant genotypes in a natural population. The markers may be suitable for selection within a breeding population where the susceptibility status of the parents is known and can be associated with a particular marker allele. Rearrangements on the proximal and distal sides of *Tsc1* relative to the Chinese Spring v2.1 reference genome is further evidence that the *Tsc1* region is a high recombination region, resulting in highly polymorphic markers, and increasing the difficulty in finding a marker that cosegregates with *Tsc1* in a natural population. These findings emphasize the need for cloning the *Tsc1* gene, which will allow the development of SNP markers based on causal polymorphisms within the gene itself and can be used to select genotypes that lack *Tsc1* using high-throughput genotyping platforms.

## Data Availability Statement

The datasets presented in this study can be found in the Supplementary Material.

## Author Contributions

KR, AM, and JF initiated and planned the study and developed markers. MA and AC developed the mapping populations. GK and JZ performed initial genotyping analyses with SNP arrays. GK and ZL performed tan spot inoculation experiments and analyses. KR performed linkage and genomic analyses. KR and JF interpreted the data and wrote the manuscript. All authors reviewed and edited the manuscript.

## Funding

This work was supported by the U.S. Department of Agriculture-Agricultural Research Service. USDA is an equal opportunity provider and employer.

## Conflict of Interest

The authors declare that the research was conducted in the absence of any commercial or financial relationships that could be construed as a potential conflict of interest.

## Publisher’s Note

All claims expressed in this article are solely those of the authors and do not necessarily represent those of their affiliated organizations, or those of the publisher, the editors and the reviewers. Any product that may be evaluated in this article, or claim that may be made by its manufacturer, is not guaranteed or endorsed by the publisher.

## Footnotes

^1^ https://wheat.pw.usda.gov/GG3/

^2^ https://archive.gramene.org/db/markers/ssrtool

^3^ https://blast.ncbi.nlm.nih.gov/Blast.cgi

^4^ https://www.ebi.ac.uk/Tools/hmmer/search/hmmscan

## Supplementary Material

Supplementary Table 1 Molecular markers developed for the saturation mapping of the Tsc1 locus.

**Nomenclature**
LP: LMPG-6 × PI626573
LouPen: Louise × Penawawa
NE: necrotrophic effector
Ptr: Pyrenophora tritici-repentis
SNP: single nucleotide polymorphism
SSR: simple sequence repeat
DH: doubled haploids
RIL: recombinant inbred line
QTL: quantitative trait locus.
